# Patient perspectives on the need for improved hearing rehabilitation: A qualitative survey study of German cochlear implant users

**DOI:** 10.3389/fnins.2023.1105562

**Published:** 2023-01-23

**Authors:** Victoria Hunniford, Robert Kühler, Bettina Wolf, Daniel Keppeler, Nicola Strenzke, Tobias Moser

**Affiliations:** ^1^Institute for Auditory Neuroscience and InnerEarLab, University Medical Center Göttingen, Göttingen, Germany; ^2^Sensory and Motor Neuroscience, Göttingen Graduate Center for Neurosciences, Biophysics, and Molecular Biosciences, Göttingen, Germany; ^3^Department of Otolaryngology, University Medical Center Göttingen, Göttingen, Germany; ^4^Auditory Neuroscience and Optogenetics Laboratory, German Primate Center, Göttingen, Germany; ^5^Cluster of Excellence “Multiscale Bioimaging: From Molecular Machines to Networks of Excitable Cells” (MBExC), University of Göttingen, Göttingen, Germany; ^6^Collaborative Research Center 889, University of Göttingen, Göttingen, Germany; ^7^Auditory Neuroscience and Synaptic Nanophysiology Group, Max Planck Institute for Multidisciplinary Sciences, Göttingen, Germany

**Keywords:** hearing rehabilitation, cochlear implant, translational research, optogenetics, gene therapy, survey study

## Abstract

**Background:**

The electrical cochlear implant (eCI) partially restores hearing in individuals affected by profound hearing impairment (HI) or deafness. However, the limited resolution of sound frequency coding with eCIs limits hearing in daily situations such as group conversations. Current research promises future improvements in hearing restoration which may involve gene therapy and optical stimulation of the auditory nerve, using optogenetics. Prior to the potential clinical translation of these technologies, it is critical that patients are engaged in order to align future research agendas and technological advancements with their needs.

**Methods:**

Here, we performed a survey study with hearing impaired, using an eCI as a means of hearing rehabilitation. We distributed a questionnaire to 180 adult patients from the University Medical Center Göttingen’s Department of Otolaryngology who were actively using an eCI for 6 months or more during the time of the survey period. Questions revolved around patients needs, and willingness to accept hypothetical risks or drawbacks associated with an optical CI (oCI).

**Results:**

Eighty-one participants responded to the questionnaire; 68% were greater than 60 years of age and 26% had bilateral eCIs. Participants expressed a need for improving the performance beyond that experienced with their current eCI. Primarily, they desired improved speech comprehension in background noise, greater ability to appreciate music, and more natural sound impression. They expressed a willingness for engaging with new technologies for improved hearing restoration. Notably, participants were least concerned about hypothetically receiving a gene therapy necessary for the oCI implant; but expressed greater reluctance to hypothetically receiving an implant that had yet to be evaluated in a human clinical trial.

**Conclusion:**

This work provides a preliminary step in engaging patients in the development of a new technology that has the potential to address the limitations of electrical hearing rehabilitation.

## Introduction

Hearing impairment (HI) is the most prevalent sensory deficit worldwide and is associated with significant socio-economic burden. Disabling HI [i.e., a pure tone threshold of greater than 35 decibels (dB) in the better hearing ear] (WHO, 2021) affects people of all age groups. One to two out of 1,000 babies are born with disabling HI (Morton and Nance, 2006), and the probability of developing HI increases with age; approximately one-third of individuals with disabling HI are over the age of 65 (Livingston et al., 2017). Unmanaged HI limits an individual’s ability to communicate and interact with others. In children, unmanaged HI affects or even prevents acquisition of vocal speech (Kral et al., 2016). Reduced social interaction due to HI in adults can be a major burden to their private and professional lives. Furthermore, HI has a massive impact on quality of life, especially in elderly patients. In the elderly HI is associated with a greater risk of cognitive decline (Loughrey et al., 2018), depression and anxiety disorders (Cosh et al., 2018; Montero-Odasso et al., 2020; Huang et al., 2022).

Causal treatments options for disabling HI–such as gene replacement or supplementation therapy, are currently being explored; yet none have achieved approval for clinical application. For mild to moderate hearing loss, reduced cochlear sensitivity can be compensated by conventional hearing aids which primarily amplify sounds. However, hearing aids do not sufficiently help in more severe cases in which sensory inner hair cells are missing or dysfunctional. In the majority of such cases, the current treatment of choice is an electrical cochlear implant (eCI) which directly stimulates the spiral ganglion neurons (SGNs). Arguably the most successful neuroprothesis, the eCI partially restores hearing and enables speech perception in the majority of the more than one million users. Despite its success, users do not experience full hearing restoration and report significant limitations in their daily lives. Specifically, users typically have difficulty understanding speech in group conversations and/or noisy and reverberating background, with complex acoustic sounds such as music, and with perception use of tonality and voice inflection (Caldwell et al., 2017).

Currently, several innovations of hearing restoration are in preclinical development. One of them is the optical cochlear implant (oCI) (Moser, 2015; Dieter et al., 2020a; Kleinlogel et al., 2020). Making use of light through optogenetics, the oCI would optically stimulate SGNs within the cochlea. Specifically, non-disease-causing viral vectors shall be introduced into the cochlea, to render SGNs light-sensitive by transgenic expression of light-activated ion channels known as Channelrhodopsins (ChR) (Hernandez et al., 2014; Duarte et al., 2018; Keppeler, 2018; Wrobel et al., 2018). Following viral transduction of the SGNs, the genetic code of the ChR is transcribed under the control of a cell-specific promoter. When ChR-expressing SGNs are exposed to light a flux of ions rush into the cells, leading to their depolarization which then induces an action potential, thus, activating the auditory pathway (Dieter et al., 2020a). As light can be better confined in space than electrical current, the oCI can make better use of the tonotopic coding of sound frequency in the mammalian cochlea and thus transmit more spectral information about sound, promising a near natural hearing perception (Hernandez et al., 2014; Dieter et al., 2019, 2020b; Keppeler et al., 2020). Preclinical evidence suggests that single dosing of a viral vector carrying a ChR under control of the neural synapsin promoter enables a stable and overall safe ChR expression (Mager et al., 2018; Bali et al., 2022). Although there is considerable experimental evidence to support its clinical promise, only an evaluation of the oCI in human participants will determine if this technology can, indeed, improve hearing in people affected by disabling HI.

It is well-documented that the translation from preclinical experiments to clinical investigation is wrought with high rates of failure, significant costs, and large timelines; which presents a contentious issue in the scientific community (Begley and Ellis, 2012; Landis et al., 2012; Chalmers et al., 2014; Ioannidis et al., 2014). Although there are many complex steps in translation, and thus, potential areas that may contribute to translational challenges, it has been suggested that engagement of patients in the research process could lead to greater chances of success in clinical trials (Duffett, 2017). Partnering with patients in medical research can help to identify research gaps and priorities, which may improve the relevance of research findings and lead to more tangible impacts in patients’ daily lives. As experts of their lived health conditions, patients can provide valuable insight into the challenges and unmet needs of the current treatment options available to them (Foster et al., 2020). Thus, in preparation for a planned first-in-human clinical trial of the oCI, we sought to identify needs and preferences regarding a new cochlear implant (CI), as well as barriers to hypothetically receiving this novel CI.

## Materials and methods

### Design

The aim of the study was to uncover patients’ preferences, expectations, and needs regarding an improved CI. From May 2020 to November 2020, questionnaires were distributed to patients of the Department of Otolaryngology of University Medical Center Göttingen (Göttingen, Germany) that were currently using a CI.

### Ethics and reporting

This study was approved through the Ethik-Kommission der Universitätsmedizin Göttingen (Protocol # 23/11/19An). The study has been reported according to the Consolidated Criteria for Reporting Qualitative Research (COREQ; Supplementary material 1) (Tong et al., 2007).

### Participants and recruitment

We recruited patients from the University Medical Center Göttingen’s Department of Otolaryngology who were actively using a CI for 6 months or more during the time of the survey period. Purposeful sampling was used to recruit participants from an internal list of patients within the Ear Clinic. Adolescent and adult patients with either unilateral or bilateral implantation were eligible for participation. Children under the age of 16 were excluded. Questionnaires were distributed and collected by post or during routine check-ups. Participation was voluntary and patients were informed that returned questionnaires implied consent. Along with the informed consent form, patients were provided with accessible information regarding the oCI; and were engaged by the research team through in-person outreach events aimed to provide them with more information regarding the oCI. Of the 81 eCI users who completed the custom questionnaire, 68% were older than 60 years.

### Questionnaire development

The custom-made questionnaire consisting of five parts and a total of 19 questions was informed by and aligned with the questionnaire items of the SSQ-12 (Speech, Spatial and Qualities of Hearing scale suitable for clinical use) (Gatehouse and Noble, 2004), as well as the IOI-HA (International Outcome Inventory for Hearing Aids) (Cox et al., 2000). The questionnaire development process consisted of the following: starting with an iterative round-table session between study investigators (including senior audiologists, preclinical scientists, and investigators with expertise with questionnaire studies) the structure of the questionnaire was outlined, and initial questions were developed. Following a feedback round, the questions and the structure was further refined. By the inclusion of the necessary experts in CIs and qualitative research, this established the face validity of the questionnaire. Following the round-table sessions, the questionnaire was piloted on additional members of the research team with experience working with CI patients. After feedback from piloting, the wording of the questions was refined for clarity and for reducing potential bias. From this, we established the content validity (final questionnaire can be found in the Supplementary material 2 for the original in German and Supplementary material 3 for the English translation). The questionnaire explored the participants’ preferences and needs regarding an improved CI; as well as willingness to accept hypothetical risks or disadvantages associated with new CI technology. Demographic information was also collected.

### Data analysis

Collected questionnaires were de-identified and assigned an identification number; responses were then entered into a Microsoft Excel spreadsheet (Microsoft Corporation, 2018). Frequencies of numerical data were calculated, and demographic data was summarized with descriptive statistics. Results were presented tabularly or graphically (GraphPad Software, Inc, 2018), where appropriate.

Written responses were inductively analyzed using thematic analysis (Braun and Clarke, 2006), where a single investigator (VH) assigned preliminary themes (or codes). All themes and codes were derived from the data. After feedback and agreement from a second investigator (TM), themes were adjusted when necessary and the coding strategy was finalized. Results from this analysis was presented narratively. The codebook can be found in the Supplementary material 4.

## Results

The survey was distributed to 180 patients in the Ear Clinic of Göttingen’s Department of Otolaryngology from May to November 2020 (approximately 6 months), and 81 participants returned a completed questionnaire (response rate of 45%). Sixty-eight percent of participants were over the age of 60 years; and 58 (72%) were unilaterally fitted with a CI (59% with left ear implanted). Of participants that had an implant in one ear, 72% wore a hearing aid in the contralateral ear and eight (14%) could hear normally in the other ear. Participant demographics and characteristics of their CI can be found in Tables 1, 2.

**TABLE 1:** Participant characteristics (*N* = 81).

Demographic characteristics		n (%)
Age	<18 years	2 (2.5)
	18–30 years	3 (4)
	30–60 years	20 (25)
	>60 years	54 (68)
	Not reported	2 (2.5)
	Unilateral	58 (72)
Cochlear implant fitting	Bilateral	21 (26)
	Not reported	2 (2.5)
**Cochlear implant use**		**Median (range)**
Participant-reported cochlear implant use per day in hours		15 (0–19)
Years with implant		3 (6 months to 25.5 years)

**TABLE 2:** Self-reported characteristics of the 58 participants that are fitted with a unilateral cochlear implant (CI) (23 participants were fitted bilaterally).

Ear that is implanted	n (%)
Left ear implanted	34 (59)
Right ear implanted	22 (38)
Not reported	2 (3)
**Characteristics of unilateral-fitted cochlear implant users**	
Normal hearing in the other ear	8 (14)
Hard of hearing in the other ear	11 (19)
Profoundly deaf or hard of hearing in the other ear	23 (40)
Use of a hearing aid in the other ear	42 (72)
No use of a hearing aid in the other ear	12 (21)
None of the statements are correct	3 (5)
No response	1 (2)

Percentage is of 58.

### Perceived importance of hypothetical improvements in a new cochlear implant

**FIGURE 1:**
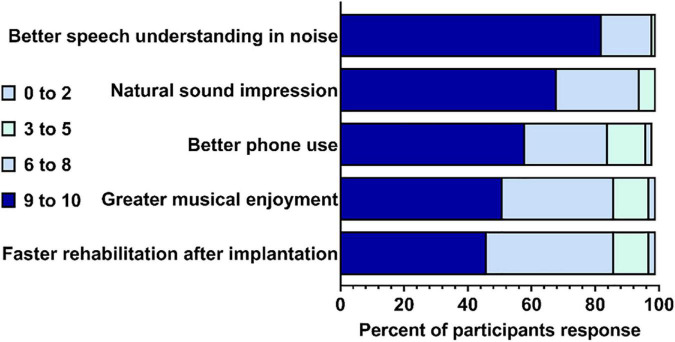
Participants’ rating of importance for five hypothetical improvements in a new cochlear implant (CI). Percent out of *N* = 80 completed responses (1 non-respondent). Gray quadrant indicates the percent of participants that rated the improvement from 0 to 2; light green indicates the percent that rated 3–5; light blue indicates that percent that rated 6–8; and dark blue indicated the percent that rated 9–10.

Participants were asked to rate hypothetical improvements in a new CI on a scale of importance from 0 to 10 (0 being not important; 10 being very important). The five improvements with a new CI [compared to what their experience(d) with their current CI] were: having faster rehabilitation after implantation, having greater enjoyment of music, having improved phone calls, having better understanding of speech in noisy environments, and more natural sound impression (Figure 1). Overall, the participants found that all five improvements were important – each item was rated as “very important” (9–10) by 46% of participants or more, and very few (no more than 3%) participants rated each item as “not important” (0 to 1). Understanding speech in background noise had the highest mean participant rating, while faster rehabilitation after implantation had the lowest (see Figure 1).

### Acceptance of potential risks and drawbacks of a new cochlear implant

**FIGURE 2:**
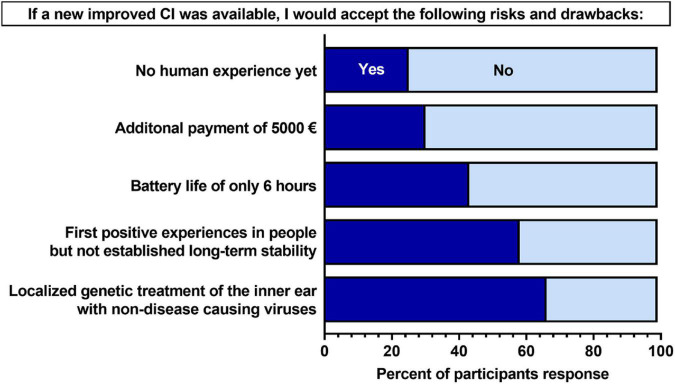
Participants’ acceptance of hypothetical risks and drawback associated with a new cochlear implant (CI). Percent values are out of the total number of participants who completed each question (*N* = 76–81). Participants were asked “If you were to be hypothetically implanted with a new CI that was expected to improve the limitations mentioned in Section I of the questionnaire (Supplementary material 2), which of the following risks and disadvantages would you accept in this new CI?” Dark blue indicates an response of “yes,” and light blue indicated a response of “no.”

Participants were asked about potential risks and drawbacks that could be associated with the novel type of CI. They were presented with five risks/drawbacks and were asked to indicate if they would accept them or not by responding with yes or no (Figure 2). For two of the five risks/drawbacks (localized genetic treatment of the inner ear with non-disease-causing viruses; first positive experiences in people, but not established long-term stability), a larger majority (50% or greater) of participants indicated “yes”–that they would accept. For the remaining three risks/drawbacks, a larger majority indicated “no”—that they would not accept. The risk/drawback which participants were least likely to accept was a lack of human experience with a new CI.

### Intentions of having a surgery for receiving a new type of cochlear implant

Participants were asked about their hypothetical intentions of having a surgery for receiving a new type of CI. They were provided with three possible responses (see Supplementary material 5). Most participants (89%) indicated that the improved CI would be an option for them, though many (33%) indicated that this would only be the case if the operation was necessary anyway. Of the nine (11%) participants who indicated “no” that the implant would not be an option for them, four provided reasoning for selecting this response. Theses participants either felt that their current CI was already sufficient, and/or they wanted to avoid the surgery for the new implant–two mentioning the elevated risks of surgery in the elderly.

### Features which participants desire in a new cochlear implant

Participants were asked what features they would want in a new CI, providing their responses as an open-text format. Fifty-four (67%) participants completed this section of the questionnaire. Responses regarding their preferences were categorized into 14 individual codes, and the codes sorted into four global themes: addressing intrinsic electrical hearing limitations; new technological features; improving practical features; and enhancing life as a CI user (Table 3). The most prevalent theme was addressing intrinsic electrical hearing limitations, and the most used code was “distinguish speech from background noise.”

**TABLE 3:** Themes and codes of participants’ open text responses to features desired in a new cochlear implant (CI).

Themes	Codes	Frequency from responses	
		Codes	Themes
Addressing intrinsic electrical hearing limitations	Distinguish speech from background noise	25	57
	Music appreciation	11	
	Understanding of speech	21	
Improving practical features	Size and comfort	18	35
	Battery life	8	
	Ease of use	5	
	No practical improvements needed	4	
New technological features	Pairing and use with other devices	8	25
	Additional technology (charging, remote control)	14	
	MRI safety, water protection	3	
Enhancing life as a CI user	Improve phone calls	8	17
	Post-op recovery and after care	5	
	Residual hearing without CI	1	
	Directional hearing	3	

The frequency that each code was used across all responses is presented along with the frequency of codes sorted into the four themes. Themes in bold represent new desired features in addition to the ones that were explored in Section I of the questionnaire (Supplementary material 2).

Of the 14 codes, 5 were areas of improvement that were already explored and surveyed in the first section of the questionnaire regarding the hypothetical improvements to a new CI (faster rehabilitation, greater enjoyment of music, improved phone calls, understanding of speech in background noise, and more natural sound impression). Thus, we uncovered nine new features that patients would desire in a new CI (bolded in Table 3). Of these new features, the most prevalent theme was improving practical features followed by new technological features. Aspects relating to size and comfort of the CI was particularly important to many participants. Several participants mentioned that they would desire a CI processor and ear hook more adapted for those who wear glasses. Several participants mentioned that they would like additional technical improvements to accompany the CI, such as the ability to charge the implant rather than replacing the batteries, a better remote control to tune the CI or to automatically adapt to the level of background noise, and more personalized programming ability. Exemplary statements from participants when asked about what features they would desire in a new CI are provided below. These reflect some of the most common themes and codes elicited from the open-text response. Additional representative statements for each of the 14 codes can be found in the Supplementary material 4.

### Statements from participants regarding features desired in a new CI

*Natural sound, better speech comprehension in the noise, better phone calls, and greater enjoyment of music.* (Participant 10)

*Better speech understanding in general, not only in direct conversation (individual, group), but also from a distance (can protrude faster). Related to this is telephoning. For sound–more balance between high and low tones. For music—the CI highlights the voice very well, but the instrumental is not yet satisfying because it does not sound so “smooth” and “fluid”*… (Participant 43)

*The environmental noise or background noise should not be so loud… It is so exhausting when you hear the air conditioning in the supermarket loudly, or the chirping of the birds… to understanding in the noise. The noise is amplified so much that you have to endure a high volume to understand [speech] as every healthy hearing person.* (Participant 4)

## Discussion

In this study, we identified CI users’ perspectives and experiences with artificial hearing as well as their preferences and willingness to try a newly developed CI. We found that with current hearing rehabilitation technologies, patients’ needs are not fully being addressed. Taken together, this work provides valuable information for the design and planning of a future early-phase clinical trial for a new CI in development—such as the optical CI.

We found that in general, participants felt that all hypothetical improvements were important. This is not surprising, as these improvements address the unmet needs and significant limitations that CI users currently experience in their day-to-day lives (Dieter et al., 2020a; Kleinlogel et al., 2020). This finding is corroborated by the ample literature documenting that CI users experience more difficulties in acoustic environments common to daily situations than their normal-hearing peers. Although much work is continuously devoted to improving the eCI, such as improved sound processing and multipolar stimulation, these advancements cannot fully address the prevailing limitations of inadequate spectral, fine-temporal, and dynamic range representation that are inherent of electrical artificial hearing (Zhou et al., 2020). This suggests that a novel modality of stimulation for artificial hearing, such as optical stimulation, would be warranted. *In vivo* and *in silico* preclinical studies confirmed the expectation that the oCI achieves near physiological (i.e., acoustic/natural hearing) spectral selectivity (Dieter et al., 2019, 2020b; Keppeler et al., 2020; Khurana et al., 2022), fundamentally exceeding that of state-of-the-art eCI, thereby promising more independent stimulation channels in the oCI than amenable to the eCI (≤10). Moreover, preclinical studies suggest an increased output dynamic range using the oCI over the eCI (Bali et al., 2021). Providing CI users with more spectral and intensity information is expected to improve the understanding of speech in multi-speaker situations as well as with fluctuating background noise, which remains a challenge with the eCI (Zheng et al., 2021). Considering these fundamental improvements to artificial hearing, it is conceivable that CI users who are unsatisfied with their current eCI would benefit from and would be open to an oCI (e.g., in the other ear not yet implanted), granted that it would indeed address the current limitations of the eCI.

Interestingly, regarding the hypothetical risks and drawbacks associated with a new CI, participants appeared to have fewer concerns about the safety-related aspects of an oCI when compared to the practical aspects. The localized genetic treatment of the inner ear with non-disease-causing viruses was the risk that the highest number of participants were accepting. Patient and public attitudes toward gene and cell therapies is an important topic, as a greater number of gene therapies continue to be explored. In a recent systematic review on patient and public perspectives on gene and cell therapies (Aiyegbusi et al., 2020), it was found that there is a general trend toward positive attitudes and acceptance of these treatment modalities; and this trend increases with the provision of information.

Regarding the practical risks and drawbacks, more than half of participants would accept the hypothetical short-coming of the oCI having higher costs and shorter battery life, respectively. These possibilities were brought to the attention of the participants, as (i) preclinical estimates of energy of pulse requirements of oCI currently exceed those of eCI and (ii) costs of oCI need to accommodate the medical device and gene therapy. Multiple participants brought up battery-life of their current eCI as an issue they would like to improve. This suggests that despite its importance to them, CI users would be more accepting of short battery time if the oCI would be an improvement in the hearing quality they experience. Fewer participants were acceptant to there being no documented human experience with the new CI yet, as clinical trials have yet to be performed. This is not surprising, since the eCI is an ambitious benchmark to surpass (Adel et al., 2019). If there has been no human experience with the oCI, it would be unknown whether it indeed demonstrates superiority to the eCI and addresses the constraints of artificial hearing that CI users must contend with. Thus, we propose that the biggest risk for patients in accepting the new oCI, would be the uncertainty that it would be an improvement to their current implant. Despite this, the majority (89%) of participants indicated that they felt the oCI would be an option for them if it were available today. This could suggest that even though there is no documented human experience, and thus, there is risk that the oCI would not be superior to their current implant, participants are optimistic that the oCI could provide them with improved hearing restoration.

Responses from the open-text section of the questionnaire provides us with valuable insight into what features CI users desire in a new CI, outside of the improvements to the well-documented limitations of artificial hearing. Notably, patients also reported practical considerations (design features, battery life, ease of use) that are of interest for improving current and new CIs–some of which are currently being considered and partially implemented in ongoing CI development ventures (Wolf et al., 2022).

### Limitations

The most notable limitation of this study is the small sampling frame, as we only included participants within a single clinic at one site. This reduces the generalizability of our study’s findings and thus, we suggest that our results should not be generalized to a larger population of individuals with HI. Moreover, the majority of the participants were over the age of 60 (68%); and have progressive hearing loss, thus, have experienced normal hearing previously in their life (data not collected in questionnaire). Therefore, is likely that the experiences and views uncovered in this study may not be applicable to younger adults and adolescents; and individuals who have prelingual hearing loss (i.e., none or limited experience with normal hearing). The large age range also adds heterogeneity to the sample population, reducing the inferential strength. Another limitation is that we included participants who had been fitted with an eCI ranging from 6 months to several years. Across our sample population, this adds large heterogeneity regarding the stage of hearing rehabilitation, thus reducing the inferential strength of our findings. Furthermore, we included participants that were fitted unilaterally and bilaterally, but did not separate these two groups in our surveying or analysis.

In addition, our results may not be reflective of non-responders to our recruitment strategy. It should also be noted that the participants in this survey have developed close patient-to-clinician relationships with some of the researchers conducting this investigation, which could bias the responses of the participants and influence the conclusions drawn from the data. Although there is low generalizability, our intention was to understand the perspectives of patients within this clinic, as this site would be the first to recruit patients for the new oCI. Building on the information elicited from the present study, a future larger survey of hearing-impaired individuals across a more diverse demographic range could confirm findings and/or identify additional views and preferences.

## Conclusion

The eCI provides a vital means for hearing rehabilitation in individuals affected by HI, however, the sensory experience of its users remains sub-optimal. Through a survey with current eCI users, we explored their experiences with artificial hearing restoration, and uncovered their preferences and opinions for a new CI. From our results, we found that in our survey population there remains an unmet clinical need in CI users’ hearing experience; and that there is openness for receiving and willingness to collaborate in the development of an oCI. Toward the development of the oCI, we will continue adopt strategies that engages patients in a meaningful way to align their preferences and needs to future research endeavors.

## Data availability statement

The raw data supporting the conclusions of this article will be made available by the authors, without undue reservation.

## Ethics statement

The studies involving human participants were reviewed and approved by Ethik-Kommission der Universitätsmedizin Göttingen. The patients/participants provided their written informed consent to participate in this study.

## Author contributions

NS and TM: supervision and resources. RK, NS, DK, and TM: conceptualization. RK, NS, VH, and TM: methodology. RK, NS, and TM: investigation. RK and VH: analysis. VH and BW: writing—original draft. All authors: writing—review and editing and approved the submitted version.
