# Supplementary material for: Patient perspectives on the need for improved hearing rehabilitation: A qualitative survey study of German cochlear implant users
Source: Front Neurosci. 2023 Jan 23;17:1105562. doi: 10.3389/fnins.2023.1105562 (PMC9899842; doi:10.3389/fnins.2023.1105562)
Supplement: Supplementary file 1 [file Data_Sheet_1.docx]

**Supplementary materials**

**Table of contents**

Supplementary material 1: COREQ (COnsolidated criteria for REporting Qualitative research) checklist

Supplementary material 2: Final original questionnaire distributed to participants (in German)

Supplementary material 3: Final questionnaire distributed to participants (translated from German to English)

Supplementary material 4: Codebook used for the thematic analysis of the open-text responses from the questionnaire

Supplementary material 5: Participants’ hypothetical intentions of having an operation for a new cochlear implant (N = 81).

**Supplementary material 1: COREQ (COnsolidated criteria for REporting Qualitative research) checklist**

| **Topic** | **Item No.** | **Guide Questions/Description** | **Reported on**  **Page No.** |
| --- | --- | --- | --- |
| **Domain 1: Research team and reﬂexivity** | | | |
| *Personal characteristics* | | | |
| Interviewer/facilitator | 1 | Which author/s conducted the interview or focus group? | NA |
| Credentials | 2 | What were the researcher’s credentials? E.g. PhD, MD | 20 |
| Occupation | 3 | What was their occupation at the time of the study? | 20 |
| Gender | 4 | Was the researcher male or female? | NA |
| Experience and training | 5 | What experience or training did the researcher have? | 20 |
| *Relationship with participants* | | | |
| Relationship established | 6 | Was a relationship established prior to study commencement? | 17 |
| Participant knowledge of  the interviewer | 7 | What did the participants know about the researcher? e.g. personal  goals, reasons for doing the research | 22/26, 17 |
| Interviewer characteristics | 8 | What characteristics were reported about the inter viewer/facilitator?  e.g. Bias, assumptions, reasons and interests in the research topic | 17 |
| **Domain 2: Study design** | | | |
| *Theoretical framework* | | | |
| Methodological orientation and Theory | 9 | What methodological orientation was stated to underpin the study? e.g. grounded theory, discourse analysis, ethnography, phenomenology,  content analysis | 7 |
| *Participant selection* | | | |
| Sampling | 10 | How were participants selected? e.g. purposive, convenience, consecutive, snowball | 6 |
| Method of approach | 11 | How were participants approached? e.g. face-to-face, telephone, mail, email | 6 |
| Sample size | 12 | How many participants were in the study? | 7 |
| Non-participation | 13 | How many people refused to participate or dropped out? Reasons? | 7 |
| *Setting* | | | |
| Setting of data collection | 14 | Where was the data collected? e.g. home, clinic, workplace | 6 |
| Presence of non-  participants | 15 | Was anyone else present besides the participants and researchers? | NA |
| Description of sample | 16 | What are the important characteristics of the sample? e.g. demographic  data, date | 6 |
| *Data collection* | | | |
| Interview guide | 17 | Were questions, prompts, guides provided by the authors? Was it pilot  tested? | 6 |
| Repeat interviews | 18 | Were repeat interviews carried out? If yes, how many? | NA |
| Audio/visual recording | 19 | Did the research use audio or visual recording to collect the data? | NA |
| Field notes | 20 | Were ﬁeld notes made during and/or after the interview or focus group? | NA |
| Duration | 21 | What was the duration of the inter views or focus group? | NA |
| Data saturation | 22 | Was data saturation discussed? | NA |
| Transcripts returned | 23 | Were transcripts returned to participants for comment and/or | NA |

**Supplementary material 2: Final original questionnaire distributed to participants (in German)**

Fragebogen „WEITERENTWICKLUNG COCHLEA IMPLANTAT“

Das Institut für Auditorische Neurowissenschaften in Göttingen arbeitet gemeinsam mit Kooperationspartnern an einer maßgeblichen Weiterentwicklung von Cochlea- Implantaten, die möglicherweise in einigen Jahren auf den Markt kommen könnten. Mit diesen Fragen möchten wir mehr über Ihre Erwartungen und Ihren Bedarf für ein verbessertes Cochlea-Implantat herausfinden.

1. **Bei welchen der folgenden Aspekte des Hörens mit Cochlea-Implantat würden Sie Verbesserungen für wichtig halten? Bitte für jeden Aspekt Punkte (von 0 – 10) vergeben!**
2. Wie wichtig ist Ihnen:

*Natürlicher Klangeindruck*

Überhaupt nicht

Sehr wichtig

0 1 2 3 4 5 6 7 8 9 10

1. Wie wichtig ist Ihnen: *Besseres Sprachverstehen im Störgeräusch*

Überhaupt nicht

Sehr wichtig

0 1 2 3 4 5 6 7 8 9 10

1. Wie wichtig ist Ihnen:

*Besseres Telefonieren*

Überhaupt nicht

Sehr wichtig

0 1 2 3 4 5 6 7 8 9 10

1. Wie wichtig ist Ihnen:

*Größerer Musikgenuß*

Überhaupt nicht

Sehr wichtig

0 1 2 3 4 5 6 7 8 9 10

1. Wie wichtig ist Ihnen:

*Schnellere Hörrehabilitation nach Implantation*

Überhaupt nicht

Sehr wichtig

0 1 2 3 4 5 6 7 8 9 10

1. **Welche der folgenden Risiken bzw. Nachteile hätten Sie in Kauf genommen, wenn wir Ihnen zum Zeitpunkt Ihrer Cochlea-Implantation ein neuentwickeltes Implantat angeboten hätten, das aus wissenschaftlich gesicherten Gründen Verbesserungen in diesen oben genannten Bereichen erwarten lässt?**

Bitte antworten Sie für die unten genannten Aspekte mit „Ja“ oder „Nein“!

| 6. Ich hätte in Kauf genommen:  *Örtlich begrenzte genetische* | - Ja  Nein | |
| --- | --- | --- |
| *Behandlung des Innenohres mit nicht-* |  |  |
| *krankmachenden Viren* |  |  |
| 7. Ich hätte in Kauf genommen: |  |  |
| *Zuzahlung von 5000 Euro* | - Ja | - Nein |
| 8. Ich hätte in Kauf genommen: |  |  |
| *Erste positive Erfahrungen am*  *Menschen, aber noch keine* | - Ja | - Nein |
| *ausreichenden Erfahrungen mit der* |  |  |
| *Langzeitstabilität* |  |  |
| 9. Ich hätte in Kauf genommen: |  |  |
| *Noch keine Erfahrungen am Menschen* | - Ja | - Nein |
| 10. Ich hätte in Kauf genommen: |  |  |
| *Eine Batterie-/Akkulaufzeit von nur*  *6 Stunden* | - Ja | - Nein |

1. **Käme heute ein solches Implantat für Sie in Frage?**
2.  Ja, auch wenn es eine erneute Operation und Rehabilitationsbehandlung erfordert.
3.  Ja, aber nur wenn ohnehin z.B. aufgrund eines Gerätedefekts eine erneute Operation notwendig wäre.
4.  Nein, weil
5. **Wenn Sie Wünsche frei hätten, welche Eigenschaften müssten zukünftige Cochlea-Implantate für Sie haben**

**Demographische Daten und Nutzungsverhalten**

1. Welcher Altersgruppe gehören Sie an?

<18 18 – 30 30 – 60 >60 Jahre

   

1. Welche Cochlea-Implantat (CI)-Versorgung besitzen Sie?
   - Einseitig  Beidseitig  Trifft nicht zu
2. Wenn EINSEITIG versorgt, welche Aussagen treffen für das andere, nicht-CI- versorgte Ohr zu?
   - Das andere Ohr hört normal gut
   - Auf dem anderen Ohr bin ich schwerhörig
   - Auf dem anderen Ohr bin ich hochgradig schwerhörig/taub
   - Ich trage auf dem anderen Ohr ein Hörgerät
   - Ich trage auf dem anderen Ohr kein Hörgerät
   - Keine der Aussagen ist richtig
3. Wie lang sind Sie schon Cochlea-Implantat-Trägerin / -Träger?

Linkes Ohr: Monate bzw. Jahre  Trifft nicht zu Rechtes Ohr: Monate bzw. Jahre  Trifft nicht zu

1. Wie lang ist Ihre durchschnittliche Tragedauer des/der CIs am Tag?

Stunden  Trifft nicht zu

**Supplementary material 3: Final questionnaire distributed to participants (translated from German to English)**

Questionnaire "FURTHER DEVELOPMENT OF COCHLEA IMPLANTS

The Institute for Auditory Neuroscience in Göttingen is working together with cooperation partners on a significant further development of cochlear implants, which could possibly come onto the market in a few years. With these questions we would like to find out more about your expectations and needs for an improved cochlear implant.

1. **In which of the following aspects of hearing with a cochlear implant would you consider improvements important? Please give points (from 0 - 10) for each aspect!**
2. How important is it to you:

*Natural sound impression*

Not at all

Very important

0 1 2 3 4 5 6 7 8 9 10

1. How important is it to you: *Better speech understanding in background noise*

Not at all

Very important

0 1 2 3 4 5 6 7 8 9 10

1. How important is it to you: *Better telephone calls*

Not at all

Very important

0 1 2 3 4 5 6 7 8 9 10

1. How important is it to you: *Greater enjoyment of music*

Not at all

Very important

0 1 2 3 4 5 6 7 8 9 10

1. How important is it to you: *Faster hearing rehabilitation after implantation*

Not at all

Very important

0 1 2 3 4 5 6 7 8 9 10

1. **Which of the following risks or disadvantages would you have accepted if, at the time of your cochlear implantation, we had offered you a newly developed implant which, for scientifically proven reasons, could be expected to improve in these above-mentioned areas?**

Please answer "Yes" or "No" for the aspects mentioned below!

| 1. I would accept:   *Localised genetic treatment of the inner ear with non-disease-causing viruses* | - Yes  No | |
| --- | --- | --- |
|  |  |  |
|  |  |  |
| 7. I would accept: |  |  |
| *Additional payment of 5000 Euros* | - Yes |  No |
| 8. I would accept: |  |  |
| *First positive experiences with*  *humans, but not yet*  *sufficient experience with the*  *long-term stability* | - Yes |  No |
|  |  |  |
| 9. I would accept: |  |  |
| *No human experience yet* | - Yes |  No |
| 10. I would accept: |  |  |
| *A battery/battery life of only*  *6 hours* | - Yes |  No |

1. **Would such an implant be an option for you today?**
2.  Yes, even if it required another operation and rehabilitation treatment.
3.  Yes, but only if a new operation would be necessary anyway, e.g. due to a device defect.
4.  No, because
5. **If it were up to you, what characteristics would you want future cochlear implants to possess?**
6. **Demographic data and usage behaviour**
7. Which age group do you belong to?

<18 18 – 30 30 – 60 >60 years

   

1. Which cochlear implant (CI) fitting do you have?
   - Unilateral  Bilateral  Does not apply
2. If fitted on one side, which statements are true for the other, non-CI fitted ear?

- I am hard of hearing in the other ear
- I am profoundly deaf/hard of hearing in the other ear.
- I wear a hearing aid in the other ear
- I do not wear a hearing aid in the other ear
- None of the statements is correct

1. How long have you been a cochlear implant user?

Left ear: Month(s) or. Year(s)  Does not apply Right ear: Month(s) or. Years  Does not apply

1. How long do you wear the CI(s) on average per day?

Hours  Does not apply

**Supplementary material 4: Codebook used for the thematic analysis of the open-text responses from the questionnaire**

| **Themes** | **Codes** | **Exemplary quote to illustrate code (quotes could be sorted into multiple codes)** |
| --- | --- | --- |
| Addressing intrinsic electrical hearing limitations | Distinguish speech from background noise | *The environmental noise or background noise should not be so loud… It is so exhausting when you hear the air conditioning in the supermarket loudly, or the chirping of the birds… to understanding in the noise. The noise is amplified so much that you have to endure a high volume to understand [speech] as every healthy hearing person.* |
|  | Music appreciation | *… music – The CI highlights the voice very well, but the instrumental is not yet satisfying because it does not sound so "smooth" and "fluid"…* |
|  | Understanding of speech | *Centrally, better speech understanding in general. Not only in direct conversation (individual, group), but also from a distance (can protrude faster).* |
| Improving practical features | Size and comfort | *The size of the processor is already very pleasant (and it is not about "hiding" by reducing the size), but the hold through the ear hook is not ideal in every situation.* |
|  | Battery life | *Battery with weekly battery life; battery for charging; Processors that are to be worn at night and that are more fixed to install* |
|  | Ease of use | *Easier to use. Especially for children.* |
|  | No practical improvements needed | *Beyond the characteristics that already exist today, no more…* |
| New technological features | Pairing and use with other devices | *… An even better connection to well-known media. Very important: More workshops/acousticians near home (not always a journey of 1-2 hours by car). More information material on the current media.* |
|  | Additional technology (charging, remote control) | *To be able to fine-tune the frequencies yourself via the Smart Phone APP or directly on the processor!* |
|  | MRI safety, water protection | *Absolute waterproof and absolute MRI suitability; Compatibility with all hearing aid brands (1 common sling for TV, mobile phone, MP3, etc); or even a direct coupling to other devices - without a sling* |
| Enhancing life as a CI user | Improve phone calls | *Better listening comprehension on the phone.* |
|  | Post-op recovery and after care | *Faster rehabilitation after surgery. Less irritation on the scalp (magnet attachment can be painful) …* |
|  | Residual hearing without CI | *Have lost the residual hearing on both ears after the CI-OP, so I am deaf when I put down the processors. I did not expect before the surgeries, or I was not informed of the risk of total hearing loss. That bothers me when I go to bed and my husband is not there. I can then not perceive any dangers associated with noise. I wish that my residual hearing could be restored through an improved CI.* |
|  | Directional hearing | *To be able to locate sounds better (shouts from other people).* |

**Supplementary material 5: Participants’ hypothetical intentions of having an operation for a new cochlear implant (N = 81).**

| ***Would this implant be an option for you today?*** | ***n (%)*** |
| --- | --- |
| Yes, even if it requires another surgery and rehabilitation. | 45 (56) |
| Yes, but only if a new operation would be necessary anyway. | 27 (33) |
| No. | 9 (11) |
